# Case series: Rare cases of thyroid storm in COVID‐19 patients

**DOI:** 10.1002/ccr3.4772

**Published:** 2021-09-05

**Authors:** Nasrin Milani, Maryam Najafpour, Masoud Mohebbi

**Affiliations:** ^1^ Department of Internal Medicine Faculty of Medicine Mashhad University of Medical Sciences Mashhad Iran; ^2^ Metabolic Syndrome Research Center Faculty of Medicine Mashhad University of Medical Sciences Mashhad Iran

**Keywords:** COVID‐19, Iran, thyroid storm, thyrotoxicosis

## Abstract

Thyroid storm is an uncommon life‐threatening thyroid dysfunction which was observed for the first time among patients infected with Coronavirus 2019 (COVID‐19). The diagnosis and treatment of the rare thyroid distinctions such as thyroid storm in COVID‐19–infected patients should be critically considered alongside common treatments of COVID‐19 infection.

## Graphical abstract

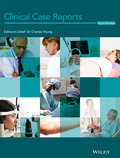

## 1 INTRODUCTION

Coronavirus disease 2019 (COVID‐19) can present pulmonary and extrapulmonary manifestations and cause multiorgan dysfunction. This is directly caused by the virus and infection of target cells, as well as its indirect damage and abnormal immune‐inflammatory responses, which lead to extensive damage and failure of various organs.^1^ Evidence suggested that COVID‐19 involves the hypothalamic‐pituitary‐thyroid axis. The inflammation and inflammatory responses caused by COVID‐19 involve the thyroid.^1^, ^2^ Based on the results of two previously conducted studies, histopathological changes have been observed in thyroid tissues of deceased patients with severe COVID‐19.^3^

In terms of thyroid function tests during COVID‐19, the results of a study on 274 COVID‐19 patients demonstrated that the concentrations of free triiodothyronine (FT3) and thyroid‐stimulating hormone (TSH) level were remarkably lower in deceased patients, as compared to those in the recovered ones (2.8 pmol/L and 0.7 mIU/ml); however, thyroxine (T4) levels did not differ significantly between the diseased and recovered patients.^4^ Other studies pointed out that in patients with COVID‐19 and severe acute respiratory syndrome‐associated coronavirus (SARS‐CoV), serum levels of T3 and TSH significantly decreased, compared to those in the control group.^5^, ^6^ The reduction in serum levels of T3 was directly related to the severity of the disease. In these patients, despite a decrease in the levels of serum T3, the serum level of TSH was reduced due to dysfunctions in the hypothalamic‐pituitary‐thyroid axis.^6^ Along the same lines, a pilot study in Pakistan indicated that 75% of patients with COVID‐19 who had thyroid abnormalities and higher interleukin‐6 (IL‐6) levels. The mentioned study suggested that changes in serum TSH and TT3 levels may be important manifestations of the courses of COVID‐19 pneumonia.^7^ After clinical recovery, these derangements in serum thyroid hormone may be permanent. Based on previous studies conducted on SARS‐CoV, 6.7% of patients developed hypothyroidism, and the majority of these patients (75%) had a central hypothyroidism.^8^

COVID‐19 cases have been presented with various manifestations of thyroid disease, including hypothyroidism, thyrotoxicosis, nonthyroidal illness syndrome,^9^ and subacute thyroiditis.^10^, ^11^ Thyrotoxicosis which has been observed during the initial phase of subacute thyroiditis and Graves’ disease can be caused by COVID‐19 as well.^9^, ^12^, ^13^ In another study, it was found that COVID‐19 could trigger autoimmune diseases, such as Graves’ disease. The stated study reported two cases of Graves’ disease after COVID‐19.^13^ Moreover, some cases of COVID‐19–associated subacute thyroiditis have been reported in literature.^10^, ^11^

The thyroid storm is a serious complication accompanied by severe clinical manifestations of thyrotoxicosis; however, it has not yet been reported in COVID‐19 cases. Nevertheless, due to the importance of thyroid storm, it is necessary to consider the coincidence of this condition in COVID‐19 patients.

## 2 CASE REPORT

On December 1, 2020, a 39‐year‐old man was referred to the hospital with a history of Graves’ disease for the past three years and lymphoblastic lymphoma since last year. He had discontinued hyperthyroidism treatment with methimazole 10 mg since last year. He had undergone four cycles of chemotherapy, and his lymphoma disease was in the remission phase. The patient presented with weakness and fatigue that became severe two weeks before hospitalization. He reported respiratory symptoms, dyspnea, diarrhea, and urinary frequency. He was also restless with warm and sweaty skin. Moreover, it is noteworthy that his eye examination results indicated proptosis. Upon admission to the hospital, he had a fever (39ºC) and tachycardia (pulse = 140). The patient had a history of contact with a COVID‐19 patient during the last two weeks before admission. Therefore, nasal swabs were collected for the COVID‐19 real‐time reverse transcription‐polymerase chain reaction (RT‐PCR) test, and the result was positive.

According to the results of the high‐resolution computed tomography, there was evidence of patchy areas of ground glass on the right side. Moreover, based on the electrocardiogram, the patient had atrial fibrillation, and his thyroid function indicated suppressed TSH (<0.05 mIU/ml; normal range: 0.35–4.95), elevated FT4(=2 pmol/L; normal range: 0.89–1.7 mg/dl), and normal FT3(=2.5 pmol/L; normal range: 1.7–2.7). The patient was evaluated based on Burch and Wartofsky Diagnostic criteria for thyroid storm^14^, ^15^, ^16^ and obtained a score of 85, including agitation (10), diarrhea (10), 39ºC fever (20), atrial fibrillation (10), tachycardia (25), and precipitant history (10). According to a previous study, central nervous system (CNS) effect and patient agitation were the only clinical features significantly different between the patients with compensated thyrotoxic and those with thyroid storm as defined by Burch‐Wartofsky scores (*p* < 0.001).^16^ In addition to the high score of diagnostic criteria which was generally affected by thyroid storm, our patient had altered mental status.

This patient underwent treatments for thyroid storm, COVID‐19, and secondary infection due to COVID‐19. These treatments included antibiotics, vancomycin, imipenem, remdesivir, anti‐thyroid (Methimazole), and steroid (hydrocortisone), beta‐blockers (propranolol), and iodine. The patient's symptoms, including heart palpitation and diarrhea relieved, as well as his level of consciousness improved after three days. Finally, he was discharged in good general health condition after eight days.

The second patient was a 50‐year‐old man with a 10‐year history of hyperthyroidism and under treatment with methimazole 5 mg. The patient experienced abdominal pain in the left flank region two weeks before admission to the hospital. The results of the initial imaging were normal, and the patient was discharged with a prescription of painkillers and Tavanex antibiotics; nevertheless, the symptoms and vomiting persisted. Moreover, diarrhea and loss of appetite were added to the symptoms. The patient gradually developed a fever, and his level of consciousness decreased as well. Eventually, on October 5, 2020, he was referred to the emergency room of Imam Reza Teaching Hospital in Mashhad, Iran, with delirium, imbalance, and urinary incontinence. He had a fever of approximately 38.5ºC and tachycardia (pulse = 100), while the results of other initial examinations were normal.

The test results are as follows: Thyroid function was assessed, showing suppressed TSH (<0.01 mIU/ml; normal range: 0.35–4.95), normal FT4 (=4 pmol/L; normal range: 1.7–3.7), and elevated FT3 (=3.1 pmol/L; normal range: 0.89–1.76). Moreover, the lung computed tomography scan and echocardiography were normal. The patient was evaluated based on the diagnostic criteria of Burch et al.^14^, ^15^, ^16^ and obtained a score of 60, including delirium (20), diarrhea, nausea, emesis, and abdominal pain (10), fever of 38.5º (15), tachycardia (5), and precipitant history (10).

On suspicion of thyroid storm, proper treatment was administrated, including hydrocortisone 100 ml, methimazole 10 mg, and propranolol 20 mg every 6 h. Given the history of contact with a positive COVID‐19 patient, a nasal swab sample was collected and examined for COVID‐19 RT‐PCR, and the result was positive. Therefore, the patient was transferred to the COVID‐19 ward for further treatment. In addition to treatment for thyroid storm, he was treated with remdesivir, interferon, and injectable antibiotics. His consciousness gradually increased during the first to sixth days of hospitalization; accordingly, the Glasgow Coma Scale increased from 9 to 14. He was able to open his eyes, express his needs, and make short conversations. Nevertheless, from the seventh day, he developed respiratory distress and underwent intubation. He was connected to a mechanical ventilator and transferred to the COVID‐19 intensive care unit; however, the patient died on the eighth day.

## 3 DISCUSSION

The patients with COVID‐19 can be asymptomatic or experience a variety of dangerous conditions.^17^ New aspects of this multiorgan disease are recognized every day. This pandemic disease has been associated with numerous autoimmune diseases, such as autoimmune thyroiditis.^10^, ^11^, ^17^ As evidenced by the SARS epidemic, a considerable number of these patients experience abnormalities in thyroid function due to virus damage to the thyroid gland and its follicular architecture.^18^ In a study performed on subjects from more than 200 countries, COVID‐19 patients were evaluated for thyroid disease. Out of 58 patients, overt thyrotoxicosis was observed in 31 (53%) cases with thyroid during the COVID‐19 pandemic. Moreover, it was found that there was a relationship between the levels of IL‐6 and TSH, suggesting inflammation‐induced damage that can exert negative effects on thyroid.^5^ In addition, the results of the aforementioned study revealed that patients with thyrotoxicosis had higher mortality rates and longer hospital stays, compared to those with normal thyroid function. Therefore, it can be stated that thyrotoxicosis has negative effects on outcomes.^5^ Furthermore, in a meta‐analysis, it was indicated that people with pre‐existing thyroid disease were more likely to develop severe COVID‐19 disease.^19^ Nevertheless, the limitations of the present study do not allow us to generalize these findings.

The current study aimed to present two cases with a history of hyperthyroidism and symptoms of a thyroid storm, along with COVID‐19 infection. Nevertheless, despite the administration of proper treatment for the thyroid storm and improvement of their symptoms, one of them eventually developed respiratory symptoms of cytokine release syndrome and died. It is noteworthy that the manifestations of COVID‐19 and thyroid storm together make the prognosis much worse. However, in previous studies, there was no evidence of thyroid storm. In fact, thyroid storm is a life‐threatening disorder, which can cause high mortality even among patients without COVID‐19 infection. It has been reported to be responsible for 10% of all deaths in Japan.^20^ It is worth noting that thyroid storm is a clinical diagnosis that can be confirmed by diagnostic criteria of Burch et al.^14^, ^15^, ^16^

Thyroid storm can be identified by different diagnostic criteria, such as Burch‐Wartofsky scores (BWSs) and Akamizu (Ak) criteria. The comparison between the diagnosis and outcomes in patients with thyroid storm and compensated thyrotoxic pointed out that patients with thyroid storm had more prolonged stays in hospital and intensive care units, ventilation requirements, and in‐patient mortality, as compared to compensated thyrotoxic patients.^16^ The reversible complications can be controlled by understanding the diagnostic criteria of thyroid storm and factors contributing to mortality in these critically ill patients who were infected with COVID‐19.

Previous studies conducted on SARS‐CoV patients illustrated that in some cases, thyroid function improved within 3–6 months, suggesting that survivors should be re‐evaluated.^8^, ^21^ According to the results of the present study, it is suggested that thyroid function tests be monitored during the acute and convalescence stages of COVID‐19. In case of any thyroid dysfunction, the possibility of replacement therapy should be considered as indicated. Furthermore, it is recommended that monitoring of thyroid function tests be continued until normal thyroid function.

## 4 CONCLUSION

It is important to consider the unknown aspects of COVID‐19 disease. These two cases confirmed that COVID‐19 may have unusual manifestations regarding thyroid storm, leading to life‐threatening conditions. Therefore, critical importance should be attached to the diagnosis and treatment of these rare cases, especially thyroid dysfunction, along with COVID‐19.

## CONFLICT OF INTEREST

The authors have no conflict of interest.

## AUTHOR CONTRIBUTIONS

NM, MM, and MN were involved with patient management and reviewed the literature. NM and MM prepared the manuscript and edited the manuscript. All authors approved the final version of the manuscript.

## ETHICAL APPROVAL

It should be noted that this study was approved by the Ethics Committee of Mashhad University of Medical Sciences (code: IR.MUMS.REC.1399.559). All the data were acquired based on the consent obtained from the patients or their guardians.

## Data Availability

Data openly available in a public repository that issues datasets with DOIs.
